# Differential Risk for Lyme Disease along Hiking Trail, Germany

**DOI:** 10.3201/eid1709.101523

**Published:** 2011-09

**Authors:** Dania Richter, Franz-Rainer Matuschka

**Affiliations:** Author affiliation: Charité Universitätsmedizin Berlin, Berlin, Germany

**Keywords:** Lyme disease, zooprophylaxis, ruminants, reservoir hosts, spirochetes, bacteria, ticks, vector-borne infections, Borrelia burgdorferi, Borrelia spp., Germany, dispatch

## Abstract

To estimate relative risk for exposure to ticks infected with Lyme disease–causing spirochetes in different land-use types along a trail in Germany, we compared tick density and spirochete prevalence on ruminant pasture with that on meadow and fallow land. Risk was significantly lower on pasture than on meadow and fallow land.

In contrast to competent hosts that serve as reservoirs for Lyme disease–causing spirochetes (hereafter called Lyme disease spirochetes), ruminants exert a zooprophylactic effect on *Borrelia burgdorferi* s.l (*1*). Fewer ticks questing on a cattle pasture harbor this pathogen than those questing elsewhere (*2*). Virtually no ticks acquire spirochetes when feeding on wild ruminants (*3*). While feeding on cattle or goats, an infected tick loses its Lyme disease spirochetes (*4*). Although wild ruminants are considered the major host of adult ticks (*5**,**6*), in the Netherlands fewer subadult ticks quested on a cattle pasture than on ungrazed sites (*7*).

It seems paradoxical that the presence of domestic ruminants reduces the density of host-seeking ticks, whereas wild ruminants appear to maintain tick populations. To determine whether ruminants used for extensive landscape management affect the exposure risk for Lyme disease for hikers, we compared tick density and spirochete prevalence along the waysides of a trail crossing a goat-and-cattle pasture, meadow, and abandoned fallow land.

## The Study

Our study site was ≈30 km west of Rothenburg-ob-der-Tauber in southern Germany. This western-facing hillside in the Jagst Valley, with an incline of 20°–40°, was originally used as vineyard, marginal fields, and grassland. After being abandoned in the late 1960s, the site was converted into 2.16-ha pasture for Limpurger cattle in 1995. Since 2003, ≈30 Bündner and Toggenburger goats have grazed with the cattle during May, September, and October. The site is enclosed by an electric fence and is characterized by a dearth of brushy vegetation. A cultural heritage hiking trail crosses the pasture and continues through former grassland that had been left to natural succession 30 years ago. A bordering meadow is mowed each July. The waysides of the trail cutting through the meadow and fallow land served as comparison with the pasture.

Questing ticks were collected by dragging a flannel flag over the ground vegetation monthly during May–October 2006 and March–October 2007. Ticks were collected per unit time by recording the period of active flagging, identified to stage and species, and preserved in 80% ethanol. To detect and identify any spirochetes infecting these ticks, we isolated DNA and amplified and sequenced a 16S rDNA fragment (*2**,**8*). For differentiation of *B. bavariensis* (spec. nov. cand [9].) from *B. garinii,* any DNA sample amplifying *B. garinii* was included in an outer surface protein A PCR and sequenced (*10*).

To estimate tick density, we collected questing ticks and extrapolated the number per hour of flagging. *Ixodes ricinus* was the sole tick species collected. On the pasture, questing ticks were least abundant (p<0.0001, Friedman test; Table 1); 12 nymphs and 2 females attached to the flag within 1 hour. On the meadow, 7× times as many questing nymphs and 5× as many females were collected in 1 hour; on the fallow land, 12× more ticks were collected. Ticks were most abundant on vegetation not modified by mowing or pasturing.

**Table 1:** Density of *Ixodes ricinus* ticks, prevalence of pathogenic Lyme disease spirochetes, and theoretic risk for exposure to such infected ticks along a trail, southern Germany, May–October 2006 and March–October 2007

Study site and tick stage	Mean no. ticks collected in 1 hour*	No. ticks examined	% Ticks infected with pathogenic spirochetes	Person’s risk for exposure in 1 hour†
Pasture				
Nymph	11.8	104	3.8‡	0.45
Adult	2.3	33	3.0§	0.07
Meadow				
Nymph	87.2	188	21.8	19.01
Adult	10.4	58	17.2	1.79
Fallow land				
Nymph	129.3	502	17.5	22.63
Adult	38.0	291	13.7	5.21

*Nymph or female. †Theoretic risk for exposure, mean number of questing nymphs or female ticks infected by pathogenic genospecies of spirochetes that cause Lyme disease: *Borrelia afzelii, B. garinii, B. bavariensis, B. burgdorferi s.s., B. spielmanii.* ‡Significantly smaller (p<0.0001 by Fisher exact test). §Comparison lacks statistical meaning because only 1 adult tick infected with pathogenic spirochetes was collected.

We determined the prevalence of spirochetes in questing nymphs and adults. Whereas only 7% of nymphs questing on pasture harbored spirochetes, 27% and 23% nymphs questing on the meadow and fallow land, respectively, contained these pathogens (p<0.0005, χ^2^ for independence; Table 2). Similarly, spirochetes were less prevalent in adult ticks questing on the pasture (6%) than on the fallow land (23%) (p<0.05, Fisher exact test). The presence of goats and cattle seems to significantly reduce spirochete prevalence in questing ticks.

**Table 2:** Prevalence of *Borrelia* genospecies in questing nymphal and adult *Ixodes ricinus* ticks sampled at different sites along a trail, southern Germany, May–October 2006 and March–October 2007

Study site and tick stage	No. ticks examined	% Ticks infected	% Ticks harboring *Borrelia* spp.*									
			*afz*	*gar*	*val*	*bur*	*lus*	*spi*	*bav*	*bis*	*miy*	>1 genospecies
Pasture												
Nymph	104	6.7	1.9	1.0	0	0	0	1.0	0	0	2.9	0
Adult	33	6.1	3.0	0	3.0	0	0	0	0	0	0	0
Meadow												
Nymph	188	26.6	17.6	2.7	0.5	0.5	2.7	1.1	0.5	0.5	1.1	0.5†
Adult	58	17.2	10.3	3.4	0	1.7	0	0	3.4	0	0	1.7†
Fallow land												
Nymph	502	22.9	11.0	5.2	4.8	0.4	0.2	1.0	0.4	0	1.6	1.6‡
Adult	291	23.4	6.9	4.5	6.9	2.7	0	0.3	0	0	2.4	0.3†

*afz, afzelii; gar, garinii; val, valaisiana; bur, burgdorferi; lus, lusitaniae; spi, spielmanii; bav, bavariensis (*9*); *bis, bissettii*-like, *miy,miyamotoi.* †Co-infection of *B. afzelii* and *B. burgdorferi* s.s. ‡Co-infection of *B. garinii* and *B. valaisiana* in 4 nymphs, *B. afzelii* and *B. spielmanii* in 2 nymphs, *B. valaisiana* and *B. lusitaniae* in 1 nymph, *B. spielmanii* and *B. miyamotoi* in 1 nymph.

We examined the spirochete genospecies infecting the ticks. On the pasture, the non–Lyme disease spirochete *B. miyamotoi* infected more nymphs than did each of the Lyme disease genospecies (Table 2). On the meadow and fallow land, *B. afzelii* was most prevalent. *B. spielmanii, B. garinii,* and *B. valaisiana* infected ticks at each site*,* whereas only *B. burgdorferi* s.s. and *B. lusitaniae* infected ticks solely on the meadow and fallow land. Ticks infected with *B. bavariensis* were collected mainly from the meadow. The prevalence of particular genospecies seems to vary with the type of landscape management.

We compared the relative risk for exposure to ticks infected with pathogenic Lyme disease spirochetes. Nymphs infected with *B. afzelii, B. garinii, B. bavariensis, B. burgdorferi* s.s., and *B. spielmanii* were significantly less prevalent on the pasture than on the meadow or fallow land (p<0.0001, Fisher exact test; Table 1). To estimate a person’s risk for exposure while walking through each of the sites for 1 hour, we multiplied the number of ticks collected within 1 hour with the rate of infection by pathogenic spirochetes. A person would have to walk through pasture for >2 hours or >14 hours to encounter a nymph or a female, respectively, infected by pathogenic spirochetes. On the meadow land, however, a person would be exposed within 1 hour to as many as 19 such infected nymphs and ≈2 females. Walking fallow land for 1 hour, a person would be exposed to >22 such infected nymphs and 5 females. Thus, risk for exposure to a questing tick infected by pathogenic spirochetes on the pasture is 40-fold and 54-fold smaller than on the meadow and fallow land, respectively.

## Conclusions

The prevalence of spirochetes in ticks questing along a hiking trail crossing a cattle-and-goat pasture, a meadow, and fallow land in southern Germany differs markedly. Where no domestic ruminants graze, ≈3.5× more ticks harbor spirochetes than on the pasture. In a previous study in the French Vosges, where cattle graze year-round on a floodplain, the zooprophylactic effect of cattle reduced prevalence of spirochetes in ticks as much as 6-fold (*2*). Not only do larvae fail to acquire spirochetes when feeding on ruminants (*3**,**11**,**12*), but previously infected ticks lose their Lyme disease spirochetes (*4*). All Lyme disease genospecies seem to be affected similarly by a tick’s blood meal on a ruminant. The prevalence of *B. miyamotoi*, however, remains unchanged independent of the presence of ruminants or mowing. With a frequency of up to 2.9% in ticks questing along the hiking trail studied, *B. miyamotoi* was as prevalent along this trail as in other sites in France, northern Germany, or southern Germany (*4**,**8**,**13*). Although domestic ruminants differentially affect these 2 kinds of spirochetes, only few ticks questing on the pasture harbor Lyme disease spirochetes.

Where domestic ruminants browse extensively, they modify the habitat. They keep the vegetation low, presumably generating a somewhat drier, less suitable microclimate for host-seeking ticks. The open habitat structure of a pasture probably displaces potential reservoir hosts by increasing their risk for predation and decreasing the quality and quantity of food (*14**,**15*). Thus, the remaining ticks on a pasture may be more likely to feed on reservoir-incompetent ruminants than on competent reservoir hosts. Fewer ticks appear to feed on rodents captured on pasture land than on those captured on woodland or grassland (*15*). This indirect effect of extensive grazing on rodent populations may amplify the zooprophylactic effect of ruminants.

Along this hiking trail, risk for exposure to ticks infected with Lyme disease spirochetes varied considerably with landscape structures. Our observations suggest that hikers are up to 54× less likely to contact a tick infected with pathogenic Lyme disease spirochetes when walking across a cattle-and-goat pasture than when walking along an abandoned area. The presence of zooprophylactic goats and cattle resulted in fewer ticks and a diminished rate of infected ticks within this local population. Extensive landscape management that uses domestic ruminants not only serves to maintain cultural and natural heritage in Germany but also seems to confer a health benefit for hikers and others seeking recreation.
